# Effect of Wine Wastes Extracts on the Viability and Biofilm Formation of* Pseudomonas aeruginosa* and* Staphylococcus aureus* Strains

**DOI:** 10.1155/2018/9526878

**Published:** 2018-07-05

**Authors:** Carolina María Viola, Romina Torres-Carro, Elena Cartagena, María Inés Isla, María Rosa Alberto, Mario Eduardo Arena

**Affiliations:** ^1^INBIOFAL (Instituto de Biotecnología Farmacéutica y Alimentaria), CONICET, Av. Kirchner 1900, Tucumán 4000, Argentina; ^2^INBIOFIV (Instituto de Bioprospección y Fisiología Vegetal), CONICET, San Lorenzo 1469, San Miguel de Tucumán 4000, Argentina; ^3^Facultad de Bioquímica, Química y Farmacia, Universidad Nacional de Tucumán, Ayacucho 491, San Miguel de Tucumán 4000, Argentina; ^4^Facultad de Ciencias Naturales e Instituto Miguel Lillo, Universidad Nacional de Tucumán, San Lorenzo 1469, San Miguel de Tucumán 4000, Argentina

## Abstract

In this work, we intended to inhibit the biofilm synthesis and the metabolism of Gram-positive and Gram-negative bacteria using two highly available wastes (stem and marc) obtained after the manufacturing of Torrontes wine at Cafayate, Argentina. Wine wastes contain a significant amount of bioactive compounds, mainly phenolic compounds, which makes them a potential source of compounds with beneficial properties to human health, as they could inhibit the virulence of pathogenic bacteria or protect the tissue against oxidative stress. Marc and stem extracts of Torrontes wine were evaluated for their ability to inhibit the metabolism and biofilm production of* Pseudomonas aeruginosa* and* Staphylococcus aureus* strains. The phytochemical composition and antioxidant activity of these extracts were also determined. The methanol and ethyl acetate extracts, which contained the highest amount of total polyphenolic, exhibited the highest scavenging capacity of ABTS and nitric oxide and the strongest Fe^3+^ reducing power and exhibited the highest level of inhibition of the biofilm formation and of the metabolic activity in bacterial biofilm. We also noticed a positive correlation between phenolic compounds content, the antioxidant activity, and the anti-biofilm capacity of the winemaking wastes. These results display the potentiality of wine wastes to prevent or reduce the formation of biofilm. Moreover, their abundance makes them an attractive and affordable source of antibiofilm and antioxidant agents.

## 1. Introduction

Biofilms are complex communities of bacteria embedded in a self-produced matrix that attach to inert or living surfaces [1]. The biofilm is an important microbial survival strategy that enables bacteria to colonize habitats, to survive to external stress, and to synchronize the bacterial population through quorum sensing to function as a multicellular organism. At the same time, it allows them to be less sensitive to antimicrobial agents [2].

Depending on the surface where the biofilm develops, it can produce cystic fibrosis, infect wounds, resulting in chronic infections that are resistant to antibiotics, or contaminate medical devices, which is the main cause of hospital infections. The biofilm formation is also an important source of concern at the food industries, since it can develop on different surfaces of the industrial machinery, becoming the principal source of foodborne diseases. In both cases, the principal concern is the impossibility of removing the biofilm using commercial cleaners or antimicrobials, since biofilm not only allows the bacterial colonization of surfaces but also enhances the bacterial community's resistance to antimicrobial agents, as well as to the host's immune system.

*Staphylococcus aureus* is a major human pathogen that is responsible for several diseases that vary from minor skin infections to life-threatening syndromes [3]. Their infective circle depends greatly on their ability to form biofilm and is the principal source of hospital infections caused by implants and the poor sanitization of medical devices [4]. Additionally,* Staphylococcus aureus* is the most found bacteria in contaminant biofilms on surfaces that are usually in contact with food, even on stainless steel [5].

*Pseudomonas aeruginosa* has been identified as the principal biofilm-forming opportunistic pathogen in chronic wounds, cystic fibrosis, as well as in several chronic infections [6, 7]. A major concern is the fact that* P. aeruginosa* produces biofilm, as well as several virulence factors, coordinately by quorum sensing when it is contaminating foods, but the addition of chemical preservatives to control biofilm is highly questioned because of their doubtful safety to human health [8]. Foodborne bacteria are a recalcitrant source of infections, and over 80% of persistent bacterial infections are associated with biofilms formed in the food manufacturing industries [9].

Many compounds are investigated as biofilm inhibitors [8]; however, only few of them are considered to be safe according to Codex Alimentarius to be used in the medicine and food industries. As a result, some essential oils and condiments have risen as potential natural inhibitors of biofilm [10, 11]. Nonetheless, these products have a high commercial cost that makes them nonprofitable to be used for this purpose. Thus, the alternative of using wastes from the food industry as biofilm inhibitors is an economically promising and environmentally friendly alternative. In this context, wine wastes present the potential to be used as biofilm inhibitors due to their richness in bioactive compounds, highlighting the possibility of producing manufactured products with a commercial benefit.

Wine production is a very important agricultural activity around the world. This industry generates a vast amount of wastes, such as grape stalks (stem), grape marc, exhausted yeast, wine lee, and high loaded wastewater [12]. The grape marc is the main component of the winery organic wastes, representing between 60 and 70% of the total disposed [13]. Typically, this waste has a high water content (ca. 60%), but on a dry basis it is comprised of skin (ca. 51%), seeds (ca. 47%), and stalks (ca. 2%) [14]. However, the specific composition of grape marc depends on the wine type, the soil, weather, and topography in which the grape is grown, as well as the winemaking techniques [15].

The industrial wastes cause a serious disposal problem and can generate pollution problems. Nevertheless, wine wastes contain bioactive compounds that might impart health benefits, opening the possibility of developing new affordable products to be incorporated into food or pharmaceutical products [16]. Various studies have been performed to explore the nature of the bioactive compounds present in these residues; the presence of polyphenols is standing out, which have been associated with the bioactive properties of grapes due to their antioxidant, anti-inflammatory, anticarcinogenic, and antibacterial activities [17–20].

On the other hand, the nature of the solvent used to extract the bioactive constituents plays an important role in the extraction process [17, 21]. Thus, using solvents of different polarities would allow a more extensive evaluation of the bioactivity of all the compounds present in the wastes. The grape's wastes are a cheap, abundant, and valuable raw material for the recovery of biologically interesting compounds. Moreover, products based on grape components are already being commercialized as supplements, as powder or capsules, which would support the safe use of compounds derived from wine wastes as part of medicinal or food products [22].

In this work, we aim to study the capacity of two wastes (stem and marc) obtained from the manufacturing of Torrontes wine at Cafayate (Argentina) to inhibit the biofilm synthesis and the metabolism of Gram-positive and Gram-negative bacteria. Moreover, numerous studies have demonstrated a strong and positive correlation between the phenolic compounds content and the antioxidant potential of grapes [23, 24]. Nevertheless, according to our knowledge, there is no information about the correlation between the antioxidant activity, the anti-biofilm capacity, and the phenolic compounds content of winemaking wastes. As a result, we also intend to determine the chemical composition of these extracts and their antioxidants properties.

## 2. Materials and Methods

### 2.1. Extractions

Wine wastes (marc and stem) were extracted using solvents of different polarities in order to obtain several extracts containing different classes of compounds. A successive extraction was developed using hexane, chloroform, ethyl acetate, and methanol. The solvent removal was performed by vacuum evaporation using a rotary evaporator (at 30°C). The soluble principle (SP) of each extract was suspended with dimethylsulfoxide (DMSO, Sigma Aldrich) for the chemical and biological analysis.

### 2.2. Phytochemical Analysis

#### 2.2.1. Determination of Total Phenolic and Nonflavonoid Compounds

Total phenolics were determined using the Folin-Ciocalteu method [25, 26]. The reaction mixture contained, in each extract, distilled water, Folin-Ciocalteu reagent, and sodium carbonate (15.9% w/v). It was maintained at 50°C for 5 min in a water bath and the absorbance was measured at 765 nm. Gallic acid was used for the standard curve (R^2^ = 0.997,* p* ≤ 0.05), and results were expressed as *μ*g gallic acid equivalents per mg of soluble principle (*μ*g GAE/mg SP).

Nonflavonoid phenolics were measured according to Torres Carro et al. [26] by quantifying the total phenolic content remaining in the supernatant after the precipitation of the flavonoids with acidic formaldehyde for 24 h. Results were expressed as *μ*g GAE/mg SP.

#### 2.2.2. Determination of Flavonoid Compounds

Total flavonoids were estimated using the method described by Popova et al. [27]. Samples were put to react with ethanol and a 5% AlCl_3_ ethanolic solution. After 30 min at room temperature, the absorbance was measured at 420 nm. Quercetin was used for the standard curve (R^2^ = 0.999,* p* ≤ 0.05), and results were expressed as *μ*g quercetin equivalents per mg of soluble principle (*μ*g QE/mg SP).

#### 2.2.3. Determination of Condensed Tannins

The total condensed tannins content was determined using 4-dimethylaminocinnamaldehyde (DMAC) according to Prior et al. [28]. Each extract, dissolved in DMSO, reacted with 0.1% DMAC and the total volume (600 *μ*L) was completed with acidified ethanol 0.1%. The mixture was put to react for 25 min at 30°C, and the absorbance was measured using a spectrophotometer at 640 nm. Proanthocyanidin B_2_ was used as standard drug, and results were expressed in *μ*g of proanthocyanidin B_2_ equivalents per mg of soluble principle (*μ*g PB_2_E/mg SP) (R^2^ = 0.989,* p* ≤ 0.05).

### 2.3. Microbiological Analysis

#### 2.3.1. Microorganisms

*Pseudomonas aeruginosa* ATCC 27853 and a strain HT5, resistant to several antibiotics, aztreonam (30 mg), ceftazidime (30 mg); cefepime (30 mg), ciprofloxacin (5 mg); gentamicin (10 mg); imipenem (10 mg), meropenem (10 mg), and piperacillin-tazobactam (110 mg), but sensitive to amikacin (30 mg), were used. The strains were grown for 24 h at 37°C in Luria-Bertani (LB) medium. In addition,* Staphylococcus aureus* ATTC 6538, and a strain HT1, methicillin-resistant, were also used. These strains were grown in Müller-Hinton (MH) medium for 24 h at 37°C.

#### 2.3.2. Bacterial Growth

Overnight cultures of both* P. aeruginosa* strains were diluted to reach an OD of 0.125 ± 0.02 at 560 nm in LB medium. Concurrently, overnight cultures of* S. aureus* strains were diluted to reach an OD of 0.13 ± 0.03 at 560 nm in MH medium. The diluted cultures (190 *μ*L) were placed in each of the 96 wells of a microtiter polystyrene plate. Solutions containing 10 and 100 *μ*g/mL of marc and stem extracts were prepared separately in DMSO/distilled water (1:1), and 10 *μ*L of each one was pipetted into the wells individually. After 24 h incubation at 37°C, bacterial growth was measured at 560 nm, using a microtiter plate reader (Multiskan Go, Thermo). A DMSO/water (1:1) solution added to the diluted culture was employed as a growth control, and the antibiotic ciprofloxacin was incorporated into the bioassay at 5 *μ*g/mL as a positive control.

#### 2.3.3. Biofilm Formation Assay

The biofilm quantification was done using a micro method based on a crystal violet stain according to a protocol previously reported [29] with several modifications [11]. Ciprofloxacin, a known biofilm inhibitor, was incorporated into the bioassay [30]. Then, the specific biofilm, which express the amount of biofilm that each bacterium forms, was calculated as the ratio between the biofilm production (measured at OD 595 nm) and the bacterial growth (measured at 560 nm) [31].

#### 2.3.4. Biofilm Metabolic Activity Assay

The metabolic activity of the biofilm formed by the bacterial strains assayed in this work was determined using a 3-[4,5-dimethylthiazol-2-yl]-2,5-diphenyltetrazolium bromide (MTT) reduction assay with some modifications [32]. Shortly, 200 *μ*L of* P. aeruginosa* and* S. aureus* cultures were incubated for 24 h at 37°C. The biofilm generated after 24 h incubation was gently removed and the plates were air-dried. Afterward, 10 *μ*L of the above-mentioned concentration of marc and stem extracts (10 and 100 *μ*g/mL) was incorporated into each well containing 190 *μ*L of PBS (pH 6.5) and was incubated for 24 h at 37°C. Then, the microplate was washed again and 100 *μ*L of MTT solution (0.5 mg/mL) was pipetted into each well and incubated for 5 h at 37°C under sterile conditions. The insoluble purple formazan salt (obtained by enzymatic hydrolysis of MTT by the dehydrogenase enzyme) was dissolved with DMSO, and the absorbance was measured at 570 nm using the microplate reader (Multiskan Go, Thermo).

### 2.4. Antioxidant Assays

#### 2.4.1. ABTS Scavenging Activity

The total antioxidant capacity of the samples was determined by the 2,2-azino-bis-(3-ehylbenzothiazoline-6-sulphonic acid) di-ammonium salt (ABTS) radical cation method as described by Torres Carro et al. [33]. An ABTS^+•^ solution (absorbance of 0.700) was added to a microplate containing the extracts (5–400 *μ*g/mL) and was mixed thoroughly. The reaction mixture was kept at room temperature for 1 min and the absorbance was immediately recorded at 734 nm. The percentage of scavenging by the samples was compared to the negative control (DMSO).

Results were expressed as percentage of ABTS^+•^ scavenging, which was calculated using the following equation:

(1) $$\begin{array}{c} \%=\left[\;\left(\;A_{0}-\frac{A_{s}}{A_{0}}\;\right)\;\right]\times 100 \end{array}$$

where A_0_ is the absorbance of the control and A_s_ is the absorbance of the samples. The percentage of scavenging was plotted as a function of concentration, obtaining the concentration of sample required to scavenge 50% of the radical (SC_50_). Quercetin (2–20 *μ*g/mL) and ascorbic acid (0.3–3 *μ*g/mL) were used as positive controls.

#### 2.4.2. Nitric Oxide Scavenging Activity

The ability of the extracts to scavenge the nitric oxide (NO) released by sodium nitroprusside under light was determined spectrophotometrically according to Torres Carro et al. [33]. Different concentrations of the extracts (200–500 *μ*g/mL) were mixed with sodium phosphate buffer (0.2 M; pH 7.4) and sodium nitroprusside (100 mM). The reaction mixture was incubated for 15 min at 37°C. Then, Griess reagent was added and the absorbance of the formed chromophore was measured at 550 nm. The SC_50_ (concentration necessary to scavenge 50% of NO) was calculated using a regression curve. Ascorbic acid was used as positive control (10–100 *μ*g/mL).

#### 2.4.3. *Fe*^+++^ to *Fe*^++^ Reducing Power

The ability of the extracts to reduce *Fe*^+++^ was assessed according to D'Almeida et al. [34]. The extract solutions (10–250 *μ*g/mL) were mixed with 416 *μ*L of 1% aqueous potassium ferricyanide and sodium phosphate buffer (0.1 M; pH 6.3) was added to reach a final volume of 1 mL. After 10 min of incubation at 50°C, 416 *μ*L of 10% trichloroacetic acid was added, and the mixture was centrifuged at 1000* xg* for 10 min. Finally, 416 *μ*L of the upper layer was mixed with 416 *μ*L of water and 83 *μ*L of 0.1% aqueous FeCl_3_. The absorbance was recorded at 700 nm after 10 min of incubation at room temperature. The percentage of reducing power was plotted against the concentration, and a linear regression analysis was carried out. The RC_50_ is the concentration necessary to reduce 50% of the Fe^3+^ and was obtained by interpolation from linear regression analysis. Ascorbic acid (2–16 *μ*g/mL) was used as positive control.

#### 2.4.4. Iron Chelating Capacity

The chelation of ferrous ions by the extracts was determined according to Torres Carro et al. [35]. Briefly, 6 *μ*L of 2 mM FeSO_4_ was added to different concentration of the extracts (100–700 *μ*g/mL) or positive control Na_2_EDTA (5–20 *μ*g/mL) and ultrapure water to a final volume of 143 *μ*L. The reaction was initiated by the addition of 7 *μ*L of 5 mM ferrozine solution which forms a colored complex with Fe^2+^. The mixture was shaken and maintained at room temperature for 10 min. The absorbance was measured using a microplate reader at 562 nm, and the percentage of inhibition of the complex formation was calculated. The chelating concentration 50% (CC_50_) is the concentration at which 50% of the iron is chelated and was obtained by interpolation from linear regression analysis.

### 2.5. Statistical Analysis

All of the assays were carried out in triplicate or quadruplicate and data are presented as mean values ± SD. The statistic software InfoStat (Student Version, 2011) was employed to evaluate the significance of differences between groups. The criterion of statistical significance was taken as* p* ≤ 0.05. The correlation studies were also analyzed using InfoStat (Student Version, 2011).

## 3. Results and Discussion

Bioactive substances are compounds characterized by their beneficial properties on human health. A natural substance is considered bioactive if it has a measurable biological activity and has a beneficial effect on health; in accordance with this, several secondary metabolites are recognized as bioactive compounds [36].

Among the most representative and well-known secondary metabolites derived from plants that have a beneficial effect on human health are the phenolic compounds. In the particular case of wine, its phytochemical composition has been extensively studied and reported. It is mostly constituted by phenolic acids, anthocyanins, flavonols, flavanols, tannins, stilbenes, etc. [37, 38]. Among the industrial byproduct from the winemaking process, grape seeds are the main component (38%–52% of dry matter) of the grape marc, whose polyphenolic composition depends mostly on the winemaking process [39]. Another important byproduct of the winemaking industry is the stem. Studies have determined that the composition of this waste consists mostly on flavonols, hydroxycinnamic acids, anthocyanins, and stilbenes [40]. For the phytochemical studies carried out in this work, we measured the most common and abundant groups of phenolic compounds present in plants to observe variations in the composition depending on the polarity of the solvent system used. As expected, a variation of the content of the different phenolic compounds was observed, increasing from the less polar solvent (hexane) to the most polar (methanol) (Table 1). The methanol extract of marc exhibited the largest proportion of polyphenols, with a content 4- to 100-folds higher than the rest of the samples. In Table 1 a variation on the proportion of the different types of polyphenols between the marc and stem extracts can also be noticed, which is probably related to a variable distribution of phenolic compounds throughout different parts of the plant. Moreover, the ethyl acetate and methanolic extracts of stem were particularly rich in nonflavonoid compounds (77 and 75% of the total, respectively). The stem extracts also showed the largest proportion of flavonoids, while the condensed tannins were more abundant in the marc samples.

Secondary metabolites like phenolic compounds are well known for their broad range of bioactivities. This property, along with their relatively safe nature, makes them an attractive target for the development of new bioproducts aimed to the cosmetic, health, and food industries.

On the other hand, industrial wastes represent an important source of pollution that lead to serious disposal problems and demand high processing costs. Therefore, it is necessary to seek for alternatives to exploit these wastes in a bid to generate a profit out of them and to reduce the amount of residues that are disposed. Wine wastes contain a vast amount of bioactive compounds known for their health benefits as antioxidants, anti-inflammatories, anticarcinogenic, etc. In accordance with this, in the present work, we evaluated the capacity of extracts of different polarities to prevent and inhibit the formation of biofilm and the growth of two bacteria that produce biofilm.

**Figure 1:**
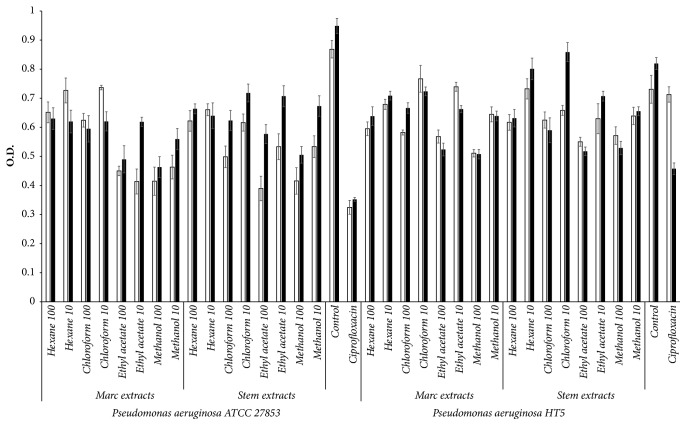
Effect of the marc and stem extracts at 10 and 100 *μ*g/mL on the viability at 570 nm (□) and biofilm production at 595 nm (■) of* Pseudomonas aeruginosa* ATCC 27853 and HT5 after 24 h of incubation. Control:* Pseudomonas aeruginosa* with the vehicle of the extracts. Data are presented as mean ± SD from three different experiments.

**Figure 2:**
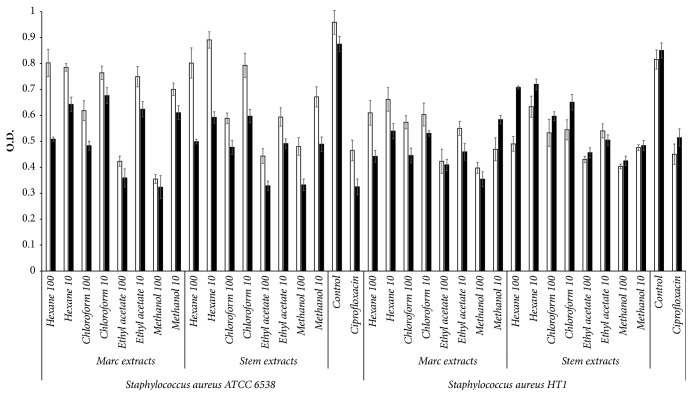
Effect of the marc and stem extracts at 10 and 100 *μ*g/mL on the viability at 570 nm (□) and biofilm production at 595 nm (■) of* Staphylococcus aureus* ATCC 6538 and HT1 after 24 h of incubation. Control:* Staphylococcus aureus* with the vehicle of the extracts. Data are presented as mean ± SD from three different experiments.

We studied the effect of the wine wastes extracts on two biofilm producing bacteria,* S. aureus* and* P. aeruginosa*, and we compared their effect on antibiotic-resistant strains and sensitive strains of both microorganisms. As shown in Figures 1 and 2, the reduction of the amount of biofilm observed appears to be due to the inhibition of biofilm production more than a depletion of viable cells, which is corroborated by the decrease noticed on the specific biofilm values. The stronger inhibition of the biofilm production was displayed by the most polar extracts of both wastes at 100 *μ*g/mL, with inhibition rates ranging from 39% to 51% for* P. aeruginosa* ATTC 27853, 35–38% for* P. aeruginosa* HT5, 59–63% for* S. aureus* ATCC 6538, and 50–58% for* S. aureus* HT1. It is important to highlight that the effect on Gram-positive strains was similar to or higher than the one observed for the control, ciprofloxacin, which exhibited a level of inhibition of 63% for S.* aureus* ATCC and 40% for HT1 (*p* ≤ 0.05). One remarkable detail was that the inhibition of the biofilm formation by the wastes was higher than their capacity to inhibit the growth of all the strains, resulting in an ability to reduce the specific biofilm, a characteristic that was not observed on the antibiotic drugs tested. Only the concentration of 10 *μ*g/mL of marc methanol extract inhibited the growth of* S. aureus* resistant strain in a similar way to the antibiotic (31%,* p* ≤ 0.05).

As for the viability of the different strains in the biofilm, the methanol and ethyl acetate extracts of marc and stem (100 *μ*g/mL) exhibited the higher levels of inhibition of* P. aeruginosa* ATTC 27853 and both strains of* S. aureus* viability (47–63% of inhibition,* p* ≤ 0.05). Moreover, a higher or equal inhibitory capacity was also observed for the* S. aureus* strains compared to the ciprofloxacin. Even though the effect observed for* P. aeruginosa *HT5 was lower (22–30%,* p* ≤ 0.05), it was still considerable taking into account the fact that ciprofloxacin did not decrease the metabolic activity of this strain in the biofilm (viability assay).

According to what was described above, we can conclude that the polar extracts of both wastes are overall more active than the less polar ones. Their major effect could be explained by their higher content of polyphenols, whose antimicrobial and antibiofilm activities have already been demonstrated [41]. Our studies have shown a positive correlation between the content of tannins in the marc extracts and their capacity to inhibit the synthesis of biofilm by* S. aureus* and* P. aeruginosa* strains (r = 0.8–0.85). While we observed a positive correlation between the content of nonflavonoids in the stem extracts and the inhibition of biofilm formation (r = 0.78–0.99), the same correlation was observed between this phenolic compound and the depletion of viability (r = 0.82–0.86) exhibited by the stem extracts on all the strains tested.

Studies have demonstrated that quorum sensing triggers cells response to oxidative stress by inducing the synthesis of scavenging enzymes [42]. It is also one of the main sources of heterogeneity in biofilm since each cell is exposed to different levels of ROS and activates their own scavenging mechanisms in response to the variable stress. ROS may also stir up adaptive mechanisms that are more effective in the biofilm environment than in a unicellular form of life and prompt dispersal of cells from biofilm [42]. Therefore, scavenging oxidative species may help to prevent biofilm formation and might explain in part the antibiofilm activity exhibited by the samples evaluated in the present work.

The antioxidant activity of phenolic compounds is one of their most well-known and studied bioactivities. A wide range of mechanisms are involved in this process and the structure of the different molecules that are joint under the group of the polyphenols is determinant. In this work, we observed that the polar polyphenols present in the methanol and ethyl acetate extracts appear to exert their antioxidant activity by scavenging radicals or by reducing metal ions. As seen in Table 2, the most active sample in scavenging the ABTS^·+^ radical was marc's methanol extract with a SC_50_ of 10.6 ± 0.4 *μ*g/mL. This sample was more than 6-fold more active than the rest of the polar extracts of both stem and marc and about 13-fold more active than chloroform fraction of stem, the only nonpolar fraction that was able to scavenge the ABTS^·+^ radical. On the other hand, the ethyl acetate extract of marc showed a similar scavenging capacity than stem's methanol and ethyl acetate extracts (Table 2). As for the iron reducing power (Table 2), the most active sample was also marc's methanol extract, with a RC_50_ 4- to 6-fold lower than the rest of the polar fractions of both samples. None of the nonpolar fractions of both samples reached the RC_50_ up to the maximum concentration tested. All the samples have a limited capacity to scavenge NO radicals and did not reach the SC_50_ values up to a maximum concentration tested (500 *μ*g/mL). Nonetheless, marc's methanol and ethyl acetate extracts were the only fractions that reached the SC_25_ up to the maximum concentration tested.

While nonpolar fractions appear to exert their antioxidant capacity mostly by chelating metal ions, as seen in Table 2, the most active fraction was the chloroform extract of stem, which was 1.6-fold more active than the rest of the nonpolar fraction, and was up to 4-6-fold more active than the methanol extracts of stem and marc. However, the only fraction that reached the SC_50_ up to the maximum concentration tested was the hexane fraction of marc (SC_50_ = 500.0 ± 3.7 *μ*g/mL), while the ethyl acetate fraction of marc was not able to chelate the Fe^2+^ at all the concentration tested. It has been proven that a specific structure is needed for a molecule to be able to chelate metals, which limits the number of molecules that exhibits this property [43]. The presence of these types of molecules would allow controlling the oxidative stress in biofilms, since reactive oxygen species (ROS) are also generated through a redox reaction led by low molecular weight iron and iron ligands [44].

Correlation studies showed a positive correlation between the iron chelating activity with the content of nonflavonoids (r = 0.98,* p* ≤ 0.05) for marc samples. On the other hand, we observed a positive correlation between the content of tannins versus ABTS scavenging capacity for marc samples (r = 0.93,* p* ≤ 0.05) and a positive correlation between the content of total phenolics (r = 0.93,* p* ≤ 0.05), nonflavonoids (r = 0.98,* p* ≤ 0.05), and tannins (r = 0.91,* p* ≤ 0.05) versus the ABTS scavenging capacity of the stem samples. There was a positive correlation between the content of total phenolic compounds (r = 0.91,* p* ≤ 0.05) and tannins (r = 0.99,* p* ≤ 0.01) versus iron reducing power in marc samples. Furthermore, there was a positive correlation with the content of nonflavonoids (r = 0.98,* p* ≤ 0.05) for the stem samples.

## 4. Conclusions

In this work, we evaluated the potential use of different extracts of grape's stem and marc of Torrontes white wine produced in Cafayate, Argentina. These byproducts inhibited the biofilm production, as well as the metabolic activity of* P. aeruginosa* and* S. aureus* strains in the biofilm environment. The major inhibition of the biofilm formation and the metabolic activity of all the strains were exerted by the polar extracts of marc and stem extracts at 100 *μ*g/mL. We also evaluated the antioxidant capacity of these extracts since it was proven that there is a correlation between oxidative stress and biofilm synthesis. The methanol and ethyl acetate extracts, which showed the highest content of polyphenolics, exhibited the strongest scavenging capacity of ABTS and NO, as well as the highest Fe^3+^ reducing power. Moreover, in accordance with these results, the correlation studies showed a positive correlation between the content of phenolic compounds, the antioxidant activity, and the antibiofilm capacity of the winemaking wastes. These results display the potentiality of wine wastes to be used to prevent or to reduce the formation of biofilm. Furthermore, their abundance makes them an attractive and affordable source of antibiofilm agents for the healthcare and food industries.

## Figures and Tables

**Table 1:** Phytochemical screening.

**Sample**	Total phenolics	Non-flavonoid phenolics	Flavonoids phenolics	Condensed Tannins
	(*μ*g GAE/mg SP)	(*μ*g GAE/mg SP)	(*μ*g QE/mg PS)	(*μ*g PB_2_E/mg SP)
**Grape Marc extract**				
Hexane	1.5 ± 0.2^a^	0.6 ± 0.1^a^	1.0 ± 0.2^a^	0.1 ± 0.1^a^
Chloroform	4.2 ± 0.2^a^	1.1 ± 0.3^a^	2.3 ± 0.1^d^	0.2 ± 0.3^a^
Ethyl acetate	33.3 ± 1.3^b^	23.0 ± 1.1^d^	1.8 ± 0.2^b,c^	54.4 ± 3.7^b^
Methanol	157.7 ± 3.1^d^	13.4 ± 0.3^c^	2.2 ± 0.1^c,d^	131.8 ± 12.9^c^

**Grape Stem extract**				
Hexane	5.6 ± 0.6^a^	1.1 ± 0.2^a^	3.7 ± 0.1^e^	0.2 ± 0.2^a^
Chloroform	25.9 ± 0.5^b^	7.0 ± 0.3^b^	4.6 ± 0.1^f^	0.8 ± 0.1^a^
Ethyl acetate	42.5 ± 4.7^c^	32.6 ± 0.3^e^	5.9 ± 0.2^g^	6.3 ± 0.1^a^
Methanol	42.1 ± 0.7^c^	31.5 ± 0.3^e^	1.7 ± 0.1^b,c^	9.7 ± 0.2^a^

GAE: gallic acid equivalents, QE: quercetin equivalents, PB_2_E: proanthocyanidin B_2_ equivalents. SP: soluble principle. Values are reported as mean ± S.D. Different letters in the same column show significant differences among each treated group, according to Tukey's test (p ≤ 0.05).

**Table 2:** Antioxidant activity of the stem and marc extracts.

**Sample**		**ABTS radical scavenging** **SC**_**50**_** (**μ**g/mL)**	**Fe** ^**3+**^ ** Reducing power** **RC**_**50**_** (**μ**g/mL)**	**Fe** ^**2+**^ ** Chelating capacity** **CC**_**25**_** (**μ**g/mL)**	**NO Scavenging capacity SC**_**25**_ ** (**μ**g/mL)**
**Marc**	**Hexane**	-	-	173.3 ± 6.9^c^	-
	**Chloroform**	-	-	172.6 ± 9.9^c^	-
	**Ehtyl acetate**	65.3 ± 0.4^c^	160.0 ± 4.7^c^	-	437.2± 0.5^c^
	**Methanol**	10.6 ± 0.4^b^	27.3 ± 0.1^a^	426.1 ± 20.9^d^	267.3± 32.4^b^

**Stem**	**Hexane**	-	-	173.5 ± 4.1^c^	-
	**Chloroform**	138.7 ± 1.9^d^	-	109.2 ± 10.3^b^	-
	**Ethyl acetate**	64.2 ± 0.9^c^	106.0 ± 1.4^b^	124.1 ± 17.9^b^	-
	**Methanol**	66.2 ± 2.5^c^	140.9 ± 1.3^b,c^	661.3 ± 2.3^e^	-

**Quercetin**		3.6 ± 0.5^a^			
**Ascorbic acid**		1.9 ± 0.4^a^	5.4± 0.03^a^		29.9 ± 0.7^a^
**Na**_**2**_ **EDTA**				5.0 ± 0.3^a^	

ABTS^·^+ radical scavenging concentration (SC), Fe3+ reducing concentration (RC), Fe2+ chelating concentration (CC), NO Scavenging capacity (SC) of marc and stem extracts. Values (mean ± SDE, n = 3) in the same column followed by the same letter are not significantly different (Tuckey's test, p ≤ 0.05).

## Data Availability

The data used to support the findings of this study are included within the article.

## Abbreviations

ABTS: 2,2-Azino-bis-(3-ehylbenzothiazoline-6-sulphonic acid) di-ammonium salt
NO: Nitric oxide
ROS: Reactive oxygen species
DMSO: Dimethyl sulfoxide
SP: Soluble principle
GAE: Gallic acid equivalents
QE: Quercetin equivalents
PB_2_E: Proanthocyanidin B_2_ equivalents
MTT: 3-[4,5-Dimethylthiazol-2-yl]-2,5- diphenyltetrazolium bromide
PBS: Phosphate buffered saline
MH: Marc hexane
MC: Marc chloroform
MA: Marc ethyl acetate
MM: Marc methanol
SH: Stem hexane
SC: Stem chloroform
SA: Stem ethyl acetate
SM: Stem methanol.
